# Modern Neuraxial Anesthesia for Labor and Delivery

**DOI:** 10.12688/f1000research.11130.1

**Published:** 2017-07-25

**Authors:** Marie-Louise Meng, Richard Smiley

**Affiliations:** 1 Department of Anesthesiology, Columbia University Medical Center, New York, NY, USA

**Keywords:** neuraxial, analgesia, epidural

## Abstract

The availability of safe, effective analgesia during labor has become an expectation for women in most of the developed world over the past two or three decades. More than 60% of women in the United States now receive some kind of neuraxial procedure during labor. This article is a brief review of the advantages and techniques of neuraxial labor analgesia along with the recent advances and controversies in the field of labor analgesia. For the most part, we have aimed the discussion at the non-anesthesiologist to give other practitioners a sense of the state of the art and science of labor analgesia in the second decade of the 21st century.

Although neuraxial (caudal and single lumbar epidural) injections were used sporadically throughout the early 20th century to reduce labor pain, the provision of labor analgesia by administration of local anesthetics via catheters inserted into the epidural space or spinal fluid is a technique that dates from the mid-20th century and did not truly become widely available until the 1980s. Over the past two decades, there have been significant advances in the quality and safety of the analgesic techniques used. The long-standing and important controversy regarding whether and how neuraxial labor analgesia affects the course of labor has been addressed and at least partially resolved. The modern era of obstetric analgesia probably should be regarded as beginning in the 1990s with the widespread adoption into clinical practice of the understanding that one could reduce local anesthetic concentrations in epidural analgesia by 40% to 60% by adding small doses of opioids (usually fentanyl or sufentanil). Other advances have included the introduction of the combined spinal-epidural (CSE) technique and, more recently, the similar “dural puncture epidural” (DPE) technique. Equipment has driven improvements too; the almost universal use of continuous infusion pumps has replaced the need for anesthesiologists to dose catheters every hour or two, which improves the obvious issue of breakthrough pain if clinician-based dosing is not timed properly. Most epidural infusion pumps now allow patient-administered top-ups (extra doses), and the newest generation of epidural pumps can be programmed to administer intermittent timed boluses (programed intermittent epidural bolus, or PIEB).

## Anatomy and physiology

Labor pain, apart from the physical component, also has emotional and cognitive elements. Pain during labor and delivery is different for every woman, and the goals of each patient’s pain relief vary, depending on how she perceives the physical, emotional, and cognitive dimensions of pain. For the parturient, satisfaction with pain relief during labor not only may be a function of relief of physical pain but also is very dependent on practitioners’ attitudes, the parturient’s expectations, and her ability to exercise some control over the delivery ^1, 2^. Interestingly, it has been reported that partners of laboring patients have less anxiety and feel more involved in the labor process if their partners receive epidural analgesia ^3^. Researchers are investigating the possibility that women who receive neuraxial pain relief in labor may be at a lower risk of post-partum depression and mood disorders ^4^.

Pain and the stress response to labor induce the release of corticotropin, cortisol, norepinephrine, β-endorphins, and epinephrine into the maternal circulation and this can result in a decrease in uterine blood flow. Pain reduction and sympathectomy caused by neural blockade result in lower levels of catecholamines and improvement in uteroplacental perfusion, especially in states of low uterine blood flow (pre-eclampsia and intrauterine growth retardation) ^5– 8^. The pain of uterine contractions and cervical dilation originates in the visceral afferent nerve fibers that enter the spinal cord at the levels of T10–L1. As the fetus descends into the vaginal canal, the somatic pain arising from the maternal pelvic floor travels along the pudendal nerve fibers S2–4. The ability to rapidly convert labor epidural analgesia to epidural surgical anesthesia and avoid general anesthesia (for mom and fetus) is generally regarded as an additional benefit of placing an epidural catheter during labor. Maternal airway anatomy changes during pregnancy, making difficult ventilation and difficult intubation real possibilities in the pregnant patient; multiple studies suggest that the incidence of failed intubation in pregnancy is much higher than in non-pregnant surgical patients ^9^. A functioning epidural catheter in a patient who may be difficult to intubate or ventilate is a safety strategy, as the catheter can be used to provide surgical anesthesia and airway manipulation can be avoided should a patient require an emergency intrapartum cesarean delivery (CD).

## Effect of presence and timing of analgesia on the course of labor

The possible effect of epidural analgesia on the course of labor has been and still is a subject of major controversy. Studies examining whether epidural analgesia (usually compared with intravenous opioids) slows labor or results in increased CD rates are very difficult to do because of ethical/consent issues and because of crossover from subjects who opt to receive epidural analgesia as “rescue” analgesia after being assigned to the non-epidural group. Still, the consensus from the studies that have been done is that if there is any effect, it is small and probably dose-dependent. The concern is that with larger doses of local anesthetic, the muscle relaxation and motor block may be greater, which may have an effect on the descent of the fetus and on the voluntary maternal expulsive efforts. Modern local anesthetic (combined with opioid) epidural techniques probably have little to no effect on CD rates ^10, 11^. A multicenter randomized controlled trial (RCT) demonstrated that there is also no increase in operative vaginal delivery with the use of low-dose epidural anesthesia ^12^. A meta-analysis comparing epidural anesthesia with intravenous opioids for labor pain revealed that epidural anesthesia prolongs the second stage of labor (time from full cervical dilation to delivery of the fetus) by only 15 to 28 minutes ^13^. Therefore, it has been well established and stated best by the American College of Obstetricians and Gynecologists and the American Society of Anesthesiologists that “In the absence of a medical contraindication, maternal request is a sufficient medical indication for pain relief during labor” ^14^.

The *timing* of administration of epidural analgesia was also controversial until recently, and women were often told that they needed to wait until the cervix was dilated 4 or 5 cm before they could receive epidural analgesia ^15^ because to have epidural analgesia earlier would slow labor and increase CD rates. The question of timing (“early” versus “later”) as opposed to use of analgesia at any time is much easier to study in an effective way. Several high-quality randomized trials performed in the first decade of the 21st century have demonstrated clearly that epidural (or CSE) analgesia provided very early in labor versus waiting for a pre-determined (4 or 5 cm) dilatation does not affect the overall course of labor and delivery. Wong *et al*. performed two RCTs, which demonstrated that in nulliparous women in either spontaneous or induced labor, early versus late initiation of neuraxial anesthesia did not increase the risk of CD or affect the time in labor ^16, 17^. Two similar studies—one in Israel ^18^ and an extremely large (>13,000 women) study from China ^19^—confirmed these results. Based on the consistent evidence above, the current consensus does appear to be that the timing of labor analgesia does not affect the mode of delivery and the course of delivery and that most women can receive analgesia upon request regardless of stage of labor ^20^.

## Technique

In the standard epidural technique, a needle is placed into the epidural space, identifying the space by a “loss of resistance” to injection of saline or air because of the low pressure in the epidural space compared with the ligamentous structures that have been traversed. A catheter is generally placed via the needle, the needle removed, and medications given via the catheter to provide analgesia or anesthesia. In the 1990s, prompted by the availability of small-gauge “pencil point” spinal needles that rarely cause post-dural puncture headaches, many practitioners started placing long, thin spinal needles through the epidural needle, to allow a medications to be given into the cerebrospinal fluid, before threading the epidural catheter that will be used once the spinal dose wears off in 60 to 120 minutes ^21^. Opioids alone (fentanyl, sufentanil, or morphine) were found to be effective for early- and mid-first-stage labor pain, providing pain relief with no associated sympathetic or motor block. A low dose of local anesthetic (bupivacaine or ropivacaine) is now usually added, providing improved analgesia particularly in women who are already in or near the second stage and experiencing the somatic pain in the sacral regions which is associated with second-stage labor ^22, 23^. Another claimed advantage of the CSE technique is that, by seeing the return of cerebral spinal fluid via the spinal needle passed through the epidural needle and just beyond its tip, the anesthesiologist can confirm objectively that the epidural needle is actually in the epidural space. This may decrease the rate of “epidural failure” from misplaced catheters ^24, 25^.

The technique of combined spinal epidural analgesia has been used broadly at academic medical centers to produce rapid onset of analgesia for labor pain and improve the spread of sacral analgesia. Widespread acceptance of the technique in the community has been limited somewhat by the complexity of the technique and requirement for the extra needle, but also by pruritus from spinal opioids and some evidence that the rapid onset or other factors in spinal analgesia may have effects on the fetal heart rate, possibly from increased rate or strength of uterine contraction ^26– 28^. A new technique, DPE, involves the performance of a CSE but without administration of an intrathecal medication dose. A recent RCT by Chau *et al.* demonstrated that the DPE provided faster onset and greater spread of sacral analgesia and less asymmetric blocks compared with a standard epidural analgesic, suggesting that the dural puncture might facilitate medication transfer intrathecally or that the performance of a dural puncture truly aids in confirmation of midline placement of epidural, leading to a greater chance of bilateral block ^29^. The DPE may become a valuable technique for the anesthesiologist looking to avoid the mild hypotension that may be caused with rapid onset of spinal analgesia, pruritis, or alterations in the fetal heart tracing but wanting to provide improved sacral analgesia and a more certain midline epidural catheter ^29^.

## Dosing

Labor analgesia is usually initiated with both dilute long-acting local anesthetic and a lipophilic opioid either in low doses through the spinal needle if a CSE is performed (for example, bupivacaine 2.5 mg with fentanyl 5 to 20 μg) or in higher dose and volume (for example, bupivacaine 0.125% 10 to 15 mL with fentanyl 100 μg) in the epidural space as an epidural “load” when epidural or DPE analgesia is performed. The optimal maintenance infusion of medication into the epidural space is a combination of dilute long-acting local anesthetic and lipophilic opioid. This combination is synergistic, improving the analgesia while minimizing the toxicity of either agent, limiting motor blockade and significant numbness ^30– 32^. With optimal epidural analgesia, patients experience mild pressure with contractions, sense rectal pressure and the urge to bear down at the start of the second stage, and maintain the motor ability to push. This can frequently be achieved with concentrations of bupivacaine or ropivacaine in the 0.0625 to 0.125% range, combined with fentanyl 2 μg/mL, at infusion rates of around 10 to 12 mL/hour. In these doses, the concentration of local anesthesia is unlikely to impact labor outcomes ^33– 35^.

Because labor pain and the effect of any given analgesia mixture and rate of infusion are unpredictable, contemporary practice frequently includes the option of patient-controlled administration, similar to the ubiquitous intravenous patent-controlled opioid administration that has been commonly used for post-operative analgesia. Patient-controlled epidural analgesia (PCEA) was first described by Gambling *et al*. ^36^ in 1988 and has become the standard approach ^37^. A button connected to the pump allows the parturient to dose herself with extra epidural infusion medication should she desire or require stronger analgesia. This PCEA results in increased maternal satisfaction, as control over one’s own analgesia is a factor that is highly valued by laboring women. Multiple recipes and strategies have been published and are used, as a wide range of acceptable doses is possible when the patient can “titrate” to what they need.

Newer epidural pumps can administer a programed volume intermittently, at a higher infusion rate than the traditional continuous infusion, facilitating medication spread in the epidural space, resulting in improved analgesia with less motor block and with less overall consumption of local anesthesia ^38– 41^. There is evidence that this mode of administration works slightly but consistently better than a continuous infusion and perhaps this is due to better spread of medication in the epidural space when larger volumes are infused over a relatively short time by the pump. This mode of dosing is called PIEB. The optimal dose volume and time interval of dosing are in early stages of investigation but are likely about 10 mL of epidural medication every 40 minutes with maternal option of periodic extra boluses ^41, 42^.

## Other analgesia options

There are few if any effective options for labor pain relief outside of neuraxial techniques. Intravenous and intramuscular opioids are still used but are well known to be relatively ineffective. The various breathing and relaxation and self-hypnotic techniques promoted over the years often as part of “natural childbirth” have some benefit but rarely provide actual analgesia. Nitrous oxide (N _2_O) has been used for decades in the United Kingdom and other countries in Europe but was almost absent from American labor suites until a few years ago. Its major purported advantage among its promoters seems to be that patients want it and perceive of it as less invasive (more “natural”) than neuraxial analgesia ^2^. Availability of N _2_O for use during labor in US hospitals is increasing, but there is significant concern that the risks of N _2_O outweigh the benefits of its use ^43^. N _2_O has minimal if any analgesic advantage over placebo and is far less effective than neuraxial anesthesia ^43^. N _2_O also produces nausea, vomiting, and dizziness, side effects that are much less common with epidural anesthesia. It is a known neurotoxic drug that is used less and less in operating rooms and has effects on DNA synthesis and repair. Occupational exposure to nurses and physicians in poorly ventilated and scavenged labor rooms is also a concern ^44, 45^. Although there has been increased discussion about its use, N _2_O is not currently used at our institution for labor analgesia.

For patients who cannot receive neuraxial anesthesia secondary to severe coagulopathy or anatomical abnormality such as scoliosis, a continuous intravenous infusion or patient-controlled administration of fentanyl or remifentanil is an option. Fentanyl is a commonly used opioid in post-operative patient-controlled analgesia (PCA) and similar PCA infusions can be used in labor with modest efficacy. Remifentanil is a synthetic, short-acting opioid that is readily hydrolyzed by plasma esterases, resulting in a short context-sensitive half-life in both mother and fetus/neonate. Because of its rapid clearance, remifentanil may be administered at doses that cause effects that would not be acceptable for a drug that lasts hours. Although it is tricky and imperfect to time boluses of medication with the onset of contraction well enough to provide analgesia during the contractions, the fast-on-and-fast-off analgesia and sedation of remifentanil make it an effective opioid for continuous infusion or PCA infusion during labor ^46– 48^. One-to-one nursing care is required for patients on remifentanil infusions to monitor closely for sedation and respiratory depression ^49^.

In summary, labor analgesia in 2017 is a far different “product” and experience than two decades ago. Most women can expect rapid, effective analgesia with limited weakness and numbness for most of labor and a degree of control over the dosing and timing of medication and can be reassured that accepting effective pain relief for labor does not impair their labor process or negatively affect its outcome.

## Abbreviations

CD, cesarean delivery; CSE, combined spinal-epidural; DPE, dural puncture epidural; PCA, patient-controlled analgesia; PCEA, patient-controlled epidural analgesia; PIEB, programed intermittent epidural bolus; N _2_O, nitrous oxide; RCT, randomized controlled trial.
